# Sclerosing angiomatoid nodular transformation (SANT) of the spleen: A case report on CT and MRI

**DOI:** 10.1259/bjrcr.20180036

**Published:** 2019-01-31

**Authors:** Raffaella Vigorito, Davide Scaramuzza, Alessandro Pellegrinelli, Alfonso Marchianò

**Affiliations:** 1 Post-graduation School in Radiodiagnostics, University of Milan, Milan, Italy; 2 Department of Radiology, Fondazione IRCCS Istituto Nazionale Tumori Milan, Milan, Italy; 3 Pathology Unit, Fondazione IRCCS Istituto Nazionale dei Tumori, Milan, Italy

## Abstract

Solid tumors of the spleen are rare, with an incidence of 0.007% in all operating and autopsy specimens. In terms of microscopic structure and function, the spleen consists of two parts: the white pulp, which plays an important role in the immune system and the red pulp, which filters the blood.Primary splenic neoplasms can be classified into lymphoid neoplasms arising from the white pulp, and vascular neoplasms which arise from the red pulp.Primary tumors arising from vascular elements include benign lesions such as hemangioma, lymphangioma and hamartoma, intermediate lesions such as hemangioendothelioma, hemangiopericytoma and littoral cell angioma as well as the frankly malignant hemangiosarcoma.It is usually difficult to distinguish a benign from a malignant lesion with preoperative imaging studies and cytological exam by fine-needle aspiration (FNA), that is not easily obtained because of the risk of bleeding.Therefore a splenectomy should be necessary for a definitive diagnosis of splenic tumors.Martel and all for the first time described the sclerosing angiomatoid nodular transformation (SANT), like a vascular lesion of the spleen, with benign clinical course consisting by altered red pulp tissue that has been entrapped by a non-neoplastic stromal proliferative process.We describe a rare case of benign splenic mass documented with FDG/PET-CT (referred as equivocal), CT and MRI.

## Case presentation

A 56-year-old female arrived to IRCCS Istituto Nazionale Tumori Milan for a further diagnostic workup and possible treatment of incidentally hypoechoic 5 cm splenic nodule, found with abdominal ultrasonography during a follow-up, for a previous stomach GIST. The contrast-enhanced ultrasonography (CEUS) report, performed in another Institution, described a lesion with centripetal early enhancement, an anechoic central component, a progressive wash-out 44 s after injection. The nodule was evaluated as “suspicious for neoplastic lesion”.

## Investigation

The patient underwent a contrast-enhanced CT, that demonstrated a solitary, round mass, with lobulated margins. On unenhanced phase the lesion had the same spleen’s density. On arterial phase it was heterogeneous, with a lively rim enhancement associated to slight hyperdensity of radiating lines. On venous and delayed phase there was progressive filling. The central scar persisted hypoenhanced in all phases.^1^

**Figure 1.**
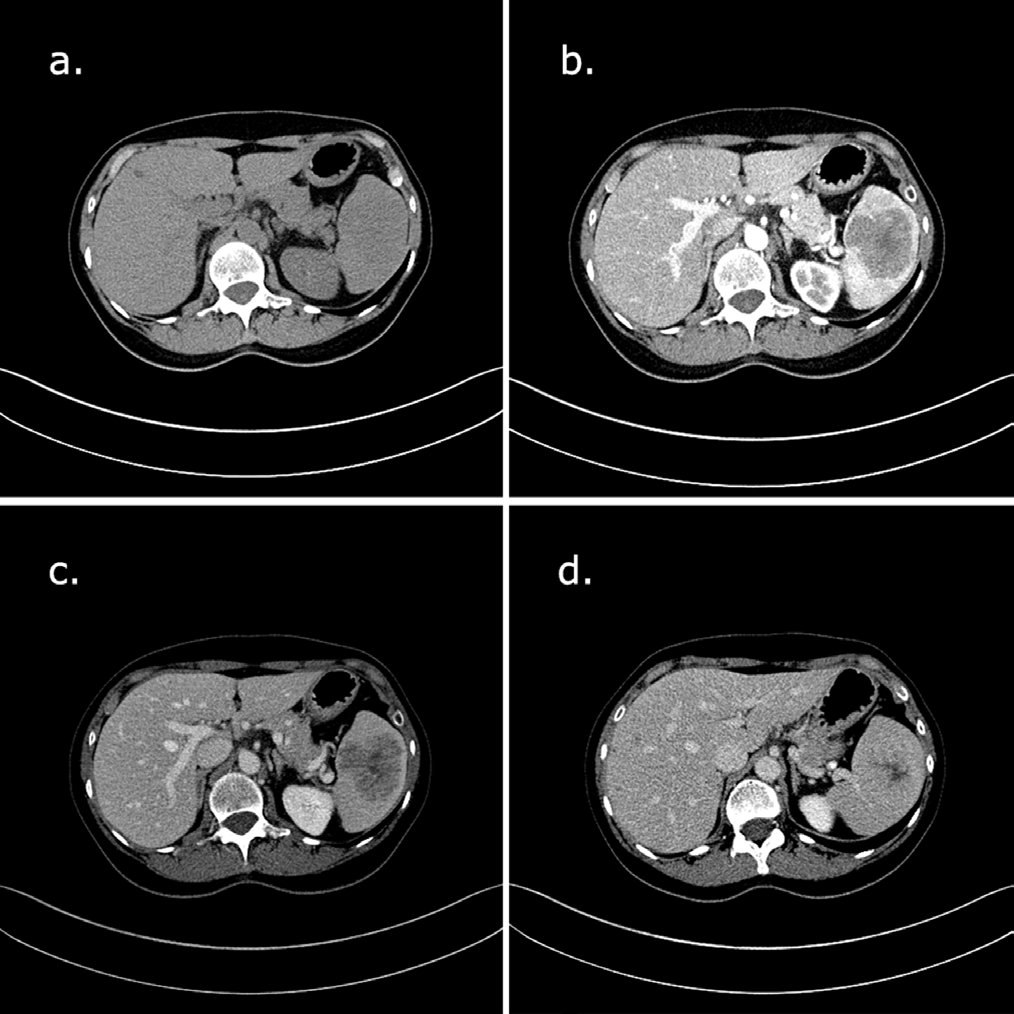
(a) On unenhanced phase the lesion has the same spleen’s density; (b) on arterial phase the lesion is rounded, with smooth margins, heterogeneous density, and lively rim enhancement associated to slight hyperdensity of radiating lines; a hypodense central scar is observed; (c–d) progressive filling on venous and delayed phase, with persisted hypoenhanced central scar.

Axial diameters were 5 × 4.5 cm and craniocaudal distance was 5.2 cm. There was no splenomegaly or lymphadenopathy (Figure 1).

**Figure 2.**
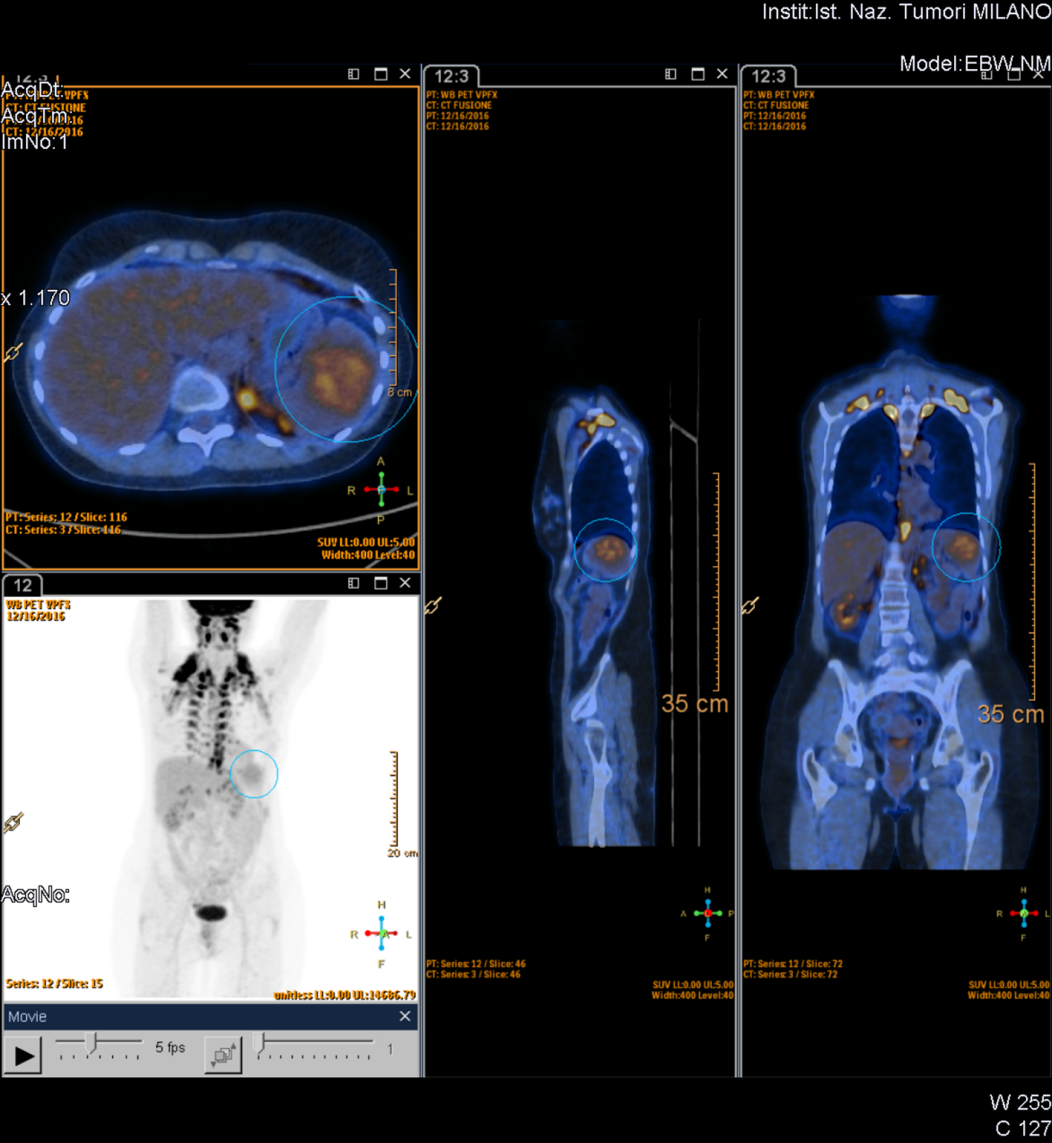
PET-CT—the lesion has low and heterogeneous FDG accumulation with a SUVmax of 4.3. FDG, fludeoxyglucose; PET, positron emission tomography; SUV_max_, maximum standardized uptake value.

Then she underwent fludeoxyglucose (FDG-PET/CT and MRI. FDG accumulation in the tumor was low and heterogeneous with a maximum standardized uptake value (SUVmax) of 4.3, referred as equivocal. No abnormal FDG accumulation has been found elsewhere in the body. Considering the CT findings, a splenic benign vascular tumor was suspected; thus, hematologist and surgeon colleagues decided to perform an MRI (Figure 2).

**Figure 3.**
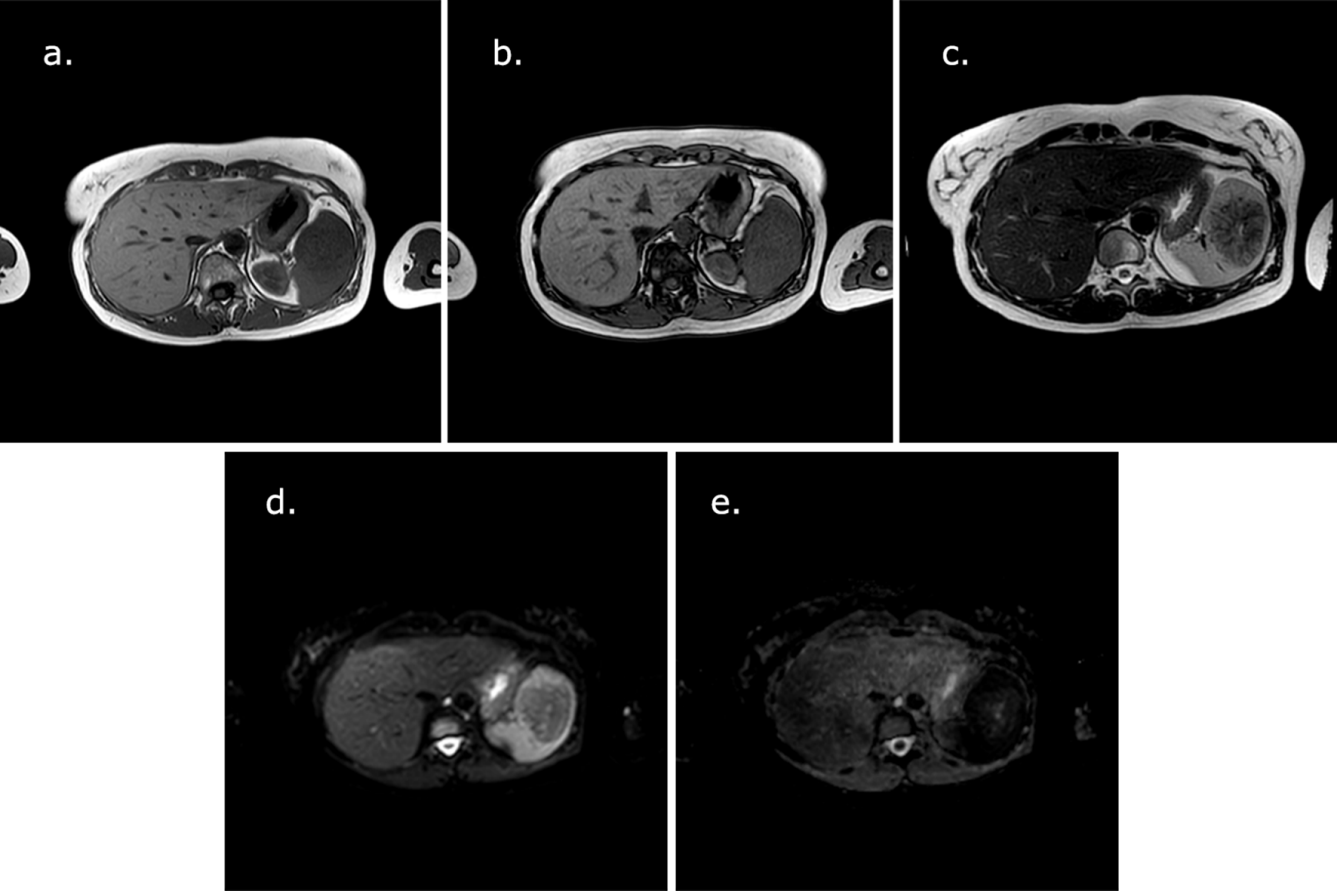
MRI pre-contrast- (a, b) the lesion isn’t clearly recognized on *T*_1_W in phase, without signal intensity drop on out of phase; (c) rounded lesion, with irregular and smooth margins, multiple radiating septa inside, with a typical “spoke wheel pattern” on *T*_1_W, *T*_1_ weighted; (d, e) absence of signal restriction (*b*-value of 800 s mm^–^^2^).

The MRI showed a 5.4 × 5.0 cm mass, with a craniocaudal diameter of 5.4 cm. Signal’s lesion was homogeneous and isointense on *T*_1_ weighted (*T*_1_W), without signal intensity drop on out-of-phase *T*_1_ sequence and inhomogeneous and predominantly decreased on *T*_2_ weighted (*T*_2_W). Morphologically the lesion was rounded, with irregular and smooth margins; there were multiple radiating septa inside, caused by the presence of a central hypointense scar. The diffusion-weighted image (DWI), with a b-value of 800 s mm^–^^2^, didn’t show signal restriction of mass, that appeared heterogeneous and reflected *T*_2_ morphology (Figure 3).

**Figure 4.**
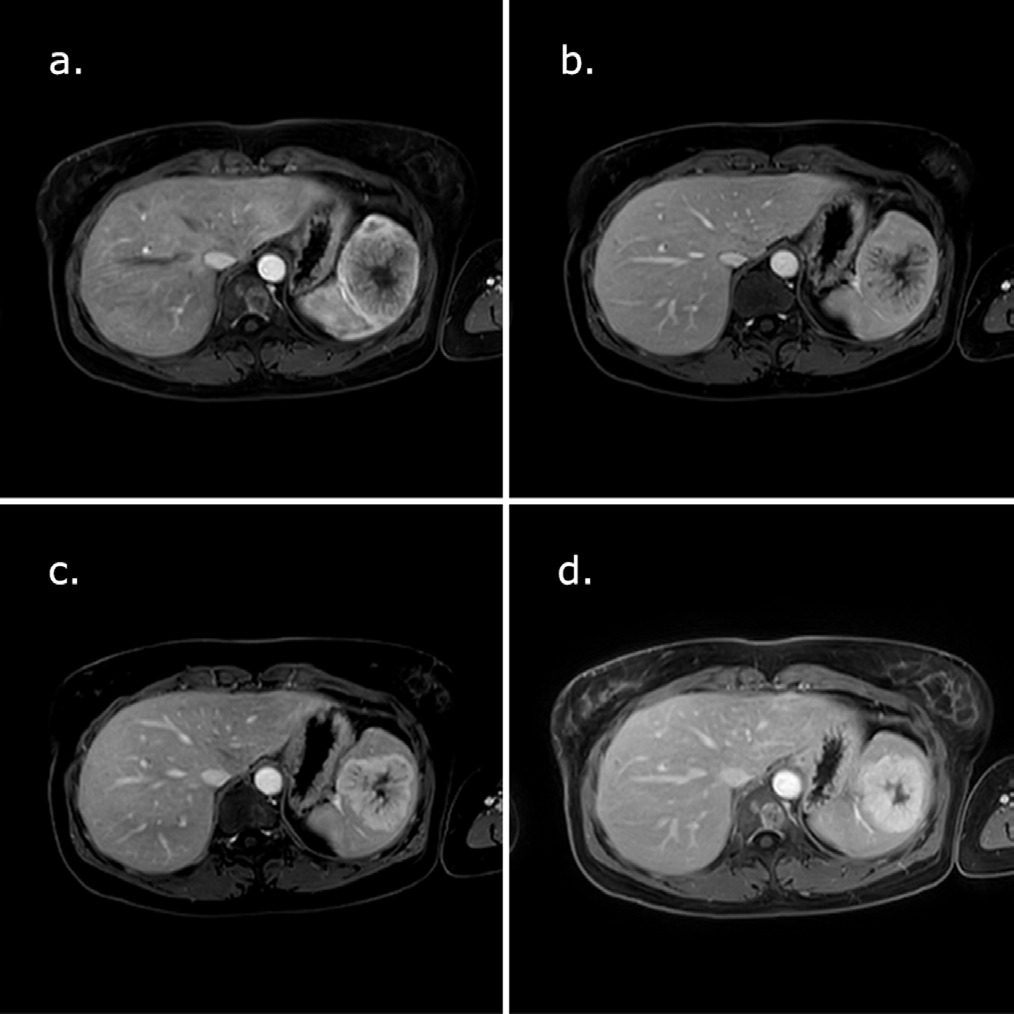
MRI after gadolinium administration: (a) on arterial phase peripheral and septa enhancement is recognizable; (b–d) on venous and delayed phase progressive and centripetal filling, with non-enhancing central hypointense scar is recognizable.

Following intravenous administration of paramagnetic contrast medium (Gadovist), on arterials phase we observed a peripheral and septa enhancement, with progressive and centripetal filling, in particular during the delayed phase, with a persistent non-enhancing central hypointense scar (Figure 4).

This lesion was considered as an hamartoma and in relation to its dimensions and superficiality (risk of bleeding) a surgical evaluation was required.

The surgeons advised for a laparoscopic/laparotomic splenectomy, which the patient refused.

Thus she has gone to 3–4 months of follow-up.

After 4 months, the MRI revealed the stability of the lesion in terms of morphology and dimension, but confirmed the surgical indication.

Finally, after 3 months a laparoscopic splenectomy was performed. At the same time, the surgeons did a partial gastrectomy for the new incidentally mesenchymal lesion of the gastric wall.

Grossly, the lesion measured 4.5 cm, it was sharply demarcated from the surrounding splenic parenchyma and was composed by coalescing red-brown nodules.

Histologically, the individual nodules had an angiomatoid comprised of round and irregular-shaped vascular spaces with extravasated red blood cells and scattered inflammatory cells. The internodular stroma consisted of variably myxoid to dense fibrous tissue with scattered plump myofibroblasts).

**Figure 5.**
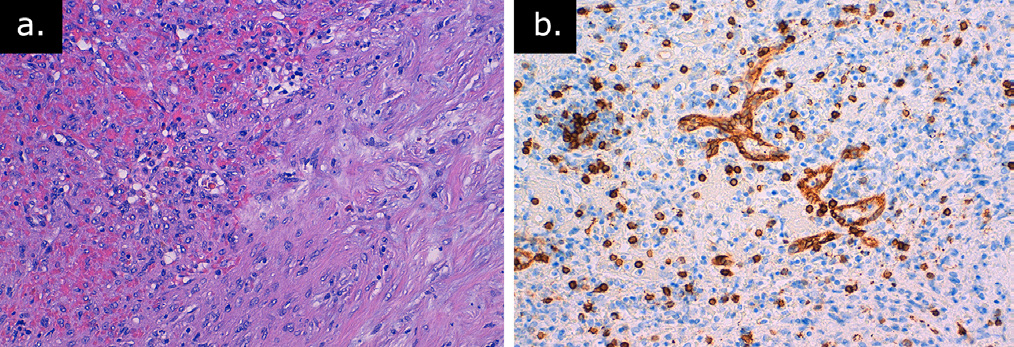
Pathology (a) angiomatoid nodule upper left, fibrous stroma lower right (EE 20 ×); (b) cd8 positive splenic sinusoid in the angiomatoid nodule (CD8 20×).

Immunostaining revealed different types of vessels in angiomatoid nodules recapitulating the composition on the normal splenic red pulp (Figure 5).

## Discussion

Sclerosing angiomatoid nodular transformation (SANT) of the spleen has been described for the first time as a distinct pathological entity in 2004 by Martel et al.

Macroscopically, SANT is a well-demarcated solitary mass composed of dense fibrous stroma containing red-brown nodules. Microscopically, the main findings are multiple angiomatoid nodules separated by collagenous bundles.^2^

SANT lesions were originally thought to occur predominantly in females. However, a recent review of the literature reports that 44.3% of the cases are males, with greater prevalence. between 30 and 60 years of age.^3^

Most lesions are asymptomatic, found incidentally on imaging, and they sometimes presents with abdominal pain.^4^

US is capable to detect vascular splenic masses, but the ultrasound findings of SANT are inconclusive,^5^ because SANT has variable appearance, variable echogenicity, and sometimes internal septa; there is usually internal vascularity on the Color Doppler.

We have found a few reports concerning contrast-enhancement behavior on CEUS; Cao at al. showed a lesion with a diffuse enhancement pattern from 11 s after injection, homogeneously hyperechoic in comparison with the splenic parenchyma in 21 s. It turned out to be isoechoic in 4 s and hypoechoic in 30 s after the injection, with a persistent enhancement up to about 7 min.^6^

A few authors have described CT and MRI findings of SANT; on CT it is solitary and round, with lobulated or smooth margins, iso- or hypoattenuating on an unenhanced scan. After medium contrast injection, the lesion shows peripheral enhancement in the arterial phase, with progressive centripetal filling in a radiating pattern, especially in the delayed phase, while its center continues to be hypodense.^7^

This “spoke wheel” pattern is recognizable also on an MRI; the lesion is usually heterogeneous, iso- to hypointense on *T*_1_, hypointense on *T*_2_. Following a gadolinium administration, it shows a peripheral and septal enhancing pattern with a hypointense central scar in all sequences; the enhancement persists on venous and delayed phase.^8^

For Yoshimura et al.^9^ DWI (*b*-value 800 s mm^–^^2^) findings correlate well with the pathological findings of SANT, the multinodular high-intensity area as the angiomatoid nodules and the peripheral low-intensity area as fibrous tissues, in both cases described.

However, in the differential diagnoses also other hypervascular focal lesions of spleen, benign or malign, must be considered.

Hamartoma on a CT appears iso- or hypodense on unenhanced phase and following contrast administration demonstrates heterogeneous enhancement. On an MRI it is isointense on *T*_1_, and heterogeneously hyperintense on *T*_2_, with heterogeneous enhancement on immediate post-contrast images, uniform and intense enhancement on delayed phase, with or without central hypovascular areas.^8^

Littoral cell angiomas of the spleen are always multiple hypoattenuating lesions with sharp margins on non-contrast CT, with an early arterial enhancement, delayed filling and eventually isointense referred to the surrounding splenic parenchyma. They are in 50% of cases hypointense on both *T*_1_- and *T*_2_ weighted images, due to hemosiderin deposition.^10^ After contrast administration, it shows mild heterogeneous enhancement on the immediate post-contrast images, with homogeneous enhancement on delayed imaging.^11^

Angiosarcoma has often bigger dimension and more aggressive behavior, with distant metastasis. On CT it can be either solitary or multiple, nodular, poorly marginated masses with heterogeneous attenuation. However, hyperattenuation can be seen in the setting of acute hemorrhage. Some lesions can show peripheral enhancement. On an MRI, these lesions are hypointense on both *T*_1_- and *T*_2_ weighted images relative to the spleen. In larger lesions hemorrhage and tumor necrosis areas can appear.^8^ These lesions demonstrate early contrast-enhancement with variable non-enhancing areas, related to necrosis or hemorrhage.

Actually differential diagnosis with only pre-operative imaging is difficult, because SANT is a rare entity and there are a few images in the literature. Moreover there is a realistic risk of rupture in cases of large spleen masses, so the current direction is splenectomy and hystological diagnosis.

In conclusion, SANT is a rare hypervascular benign lesion, recently classified as an independent entity. Knowledge of the typical “spoke wheel” aspect, and of contrast-enhanced pattern, recognizable on CT or MRI, can be helpful in a preoperative diagnosis, avoiding histological examination. A good diagnostic accuracy of DWI MRI imaging is also reported.

## Learning points

1. SANT of the spleen is a distinct benign pathological entity, microscopically characterized by multiple angiomatoid nodules separated by collagenous bundles.
2. At diagnostic examinations SANT morphology can mimic other benign or malignant splenic lesions.
3. Typical morphology and contrast-enhancement behavior on CT and MRI can be helpful for radiologic diagnosis. SANT is usually a solitary, rounded, with lobulated or smooth margins, multiple radiating septa inside, caused by the presence of hypodense/hypointense a central scar. Following intravenous administration of a contrast medium on arterial phase a peripheral and septa enhancement has been observed, with progressive and centripetal filling, particularly during the delayed phase, with a persistent non-enhancing central scar.

## References

[1] Moriyama S , Inayoshi A , Kurano R . Inflammatory pseudotumor of the spleen: report of a case . Surg Today 2000 ; 30 : 942 – 6 . doi: 10.1007/s005950070051 11059739

[2] Martel M , Cheuk W , Lombardi L , Lifschitz-Mercer B , Chan JK , Rosai J . Sclerosing angiomatoid nodular transformation (SANT): report of 25 cases of a distinctive benign splenic lesion . Am J Surg Pathol 2004 ; 28 : 1268 – 79 . 15371942 10.1097/01.pas.0000138004.54274.d3

[3] Falk GA , Nooli NP , Morris-Stiff G , Plesec TP , Rosenblatt S . Sclerosing Angiomatoid nodular transformation (SANT) of the spleen: case report and review of the literature . Int J Surg Case Rep 2012 ; 3 : 492 – 500 . doi: 10.1016/j.ijscr.2012.06.003 22858789 PMC3421142

[4] Atas H , Bulus H , Akkurt G , Ozkan H . Sclerosing Angiomatoid nodular transformation of the spleen: an uncommon cause of abdominal pain . Euroasian J Hepatogastroenterol 2017 ; 7 : 89 – 91 . doi: 10.5005/jp-journals-10018-1221 29201782 PMC5663784

[5] Wang HL , Li KW , Wang J , Hl W , KW LI . Sclerosing angiomatoid nodular transformation of the spleen: report of five cases and review of literature . Chin Med J 2012 ; 125 : 2386 – 9 . 22882867

[6] Cao J-Y , Zhang H , Wang WP . Ultrasonography of sclerosing angiomatoid nodular transformation in the spleen . World J Gastroenterol 2010 ; 16 : 3727 – 30 . doi: 10.3748/wjg.v16.i29.3727 20677348 PMC2915436

[7] Karaosmanoglu DA , Karcaaltincaba M , Akata D . CT and MRI findings of sclerosing angiomatoid nodular transformation of the spleen: spoke wheel pattern . Korean J Radiol 2008 ; 9 Suppl : S52 – 5 . doi: 10.3348/kjr.2008.9.s.s52 18607127 PMC2627191

[8] Bowerson M , Menias CO , Lee K , Fowler KJ , Luna A , Yano M , et al . Hot spleen: hypervascular lesions of the spleen . Abdom Imaging 2015 ; 40 : 2796 – 813 . doi: 10.1007/s00261-015-0523-8 26384825

[9] Yoshimura N , Saito K , Shirota N , Suzuki K , Akata S , Oshiro H , et al . Two cases of sclerosing angiomatoid nodular transformation of the spleen with gradual growth: usefulness of diffusion-weighted imaging . Clin Imaging 2015 ; 39 : 315 – 7 . doi: 10.1016/j.clinimag.2014.10.015 25457575

[10] Abbott RM , Levy AD , Aguilera NS , Gorospe L , Thompson WM . From the archives of the AFIP: primary vascular neoplasms of the spleen: radiologic-pathologic correlation . Radiographics 2004 ; 24 : 1137 – 63 . doi: 10.1148/rg.244045006 15256634

[11] Falk S , Stutte HJ , Frizzera G . Littoral cell angioma. A novel splenic vascular lesion demonstrating histiocytic differentiation . Am J Surg Pathol 1991 ; 15 : 1023 – 33 . 1928554

